# Impact of sugar-sweetened beverage tax on dental caries: a simulation analysis

**DOI:** 10.1186/s12903-020-1061-5

**Published:** 2020-03-18

**Authors:** Nipaporn Urwannachotima, Piya Hanvoravongchai, John Pastor Ansah, Piyada Prasertsom, Victoria Rui Ying Koh

**Affiliations:** 1 Department of Community Dentistry, Faculty of Dentistry, Chulalongkorn University, Bangkok, Thailand; 2 Department of Preventive and Social Medicine, Faculty of Medicine, Chulalongkorn University, Bangkok, Thailand; 3 Health Services and Systems Research, Duke-NUS Medical School, Singapore, Singapore; 4 Bureau of Dental Health, Department of Health, Ministry of Public Health, Nonthaburi, Thailand; 5 Saw Swee Hock School of Public Health, National University of Singapore, Lower Kent Ridge Road, Singapore, Singapore

**Keywords:** Dental caries, Dental public health, Sugar-sweetened beverage tax, Sugar consumption, Computer simulation, System dynamics model, System science

## Abstract

**Background:**

The tiered sugar-sweetened beverage (SSB) tax was implemented in Thailand to encourage industries to reduce sugar content in beverages, and consequently reduce sugar consumption in the population. The aim of the study is to explore the expected impact of the new SSB tax policy in Thailand, a middle-income country in Asia, and other alternative policies on oral health outcomes as measured by the prevalence and severity of dental caries among the Thai population.

**Methods:**

A qualitative system dynamics model that captures the complex interrelationships among SSB tax, sugar consumption and dental caries, was elicited through participatory stakeholder engagement. Based on the qualitative model, a quantitative system dynamics model was developed to simulate the SSB tax policy and other alternative scenarios in order to evaluate their impact on dental caries among Thai adults from 2010 to 2040.

**Results:**

Under the base-case scenario, the dental caries prevalence among the Thai population 15 years and older, is projected to increase from 61.3% in 2010 to 74.9% by 2040. Implementation of SSB tax policy is expected to decrease the prevalence of dental caries by only 1% by 2040, whereas the aggressive policy is projected to decrease prevalence of dental caries by 21% by 2040.

**Conclusions:**

In countries where a majority of the sugar consumed is from non-tax sugary food and beverages, especially Asian countries where street food culture is ubiquitous and contributes disproportionately to sugar intake, SSB tax alone is unlikely to have meaningful impact on oral health unless it is accompanied with a comprehensive public health policy that aims to reduce total sugar intake from non-SSB sources.

## Background

Despite advances in dental treatment, dental caries is still a major public health problem worldwide, especially among disadvantaged groups in both developing and developed countries. The presence of dental caries has been found to significantly affect oral health related quality of life and can lead to the eventual loss of teeth [1]. Symptoms of dental caries can not only lead to psychological stress [2] but also decrease school or work productivity of both young and older adults [3]. Current evidence supports the association between high quantity and frequent sugar consumption and high prevalence of dental caries [4, 5].

Sugar consumption in Thailand has increased significantly from 12.7 kg per capita per year in 1983 to 38.2 kg per capita per year in 2015 [6, 7]. The amount of direct sugar consumption from refined table sugar has been higher than the indirect consumption from processed food and beverages in the past 20 years [6]. However, the trend of direct sugar consumption has been declining, while that of the indirect sugar consumption is increasing [6]. Total sugar consumption in Thailand has exceeded the WHO recommendation of 10% of total energy intake of 50 g of sugar per day and below 5% of total energy intake, which is 25 g per day for additional health benefits. Furthermore, sugar consumption in Thailand has increased from an average of 76.19 g per day in year 2000 to 104.46 g per day by 2015 [7]. The fraction of total sugar consumed in Thailand from SSB sources has increased from 15% in 2000 to 21% by 2015, while that from non-SSB sources has decreased from 85% in 2000 to 79% by 2015 [7]. A review of national surveys and studies suggests that common food sources of sugar and indirect sugar consumption in all age groups were sweetened beverages, Thai desserts, and confectionery [8]. Among these food sources, sugar-sweetened beverages (SSB) represent the largest source of sugar consumption [8, 9]. In the last few decades, SSB consumption has been on the rise in many parts of the world including Thailand [6, 10]. Twenty four percent of the Thai population consumed at least one serving of SSB daily, which contains sugar ranging from 10 g in dairy product and cereal drinks, to 34 g per serving in soft drinks [9, 11].

In an effort to reduce sugar consumption, an SSB tax policy has been implemented by several countries such as Hungary [12], France [13], Mexico [14], and some cities in the United States [15]. Recently, Australia, The Philippines [16], UK [17], India and South Africa [18] have taken the initiative to implement a similar SSB tax policy. As evidence suggest that SSB consumption decreases when price increases, leading to a reduction in average daily calorie intake and BMI [17, 19, 20].

In 2017, the Thai Excise Department implemented a tiered SSB tax policy. The SSB tax was implemented because sugar consumption in Thailand has exceeded the WHO recommendation and due to the increasing prevalence of health problems linked to high sugar consumption, such as obesity (the prevalence of individuals with BMI ≥30 kg/m^2^ has increased from 12.1% in 2004 to 19.8% in 2014 [21]), diabetes (prevalence of diabetes has increased from 7.1% in year 2004 to 10.85% in year 2014 among Thai population aged 20 years or older [21]), hypertension (prevalence of hypertension among the Thai population aged 20 years and older has increased from 45% in 2004 to 48.1% in 2014 [21]), and dental caries (prevalence of dental caries has increased from 85.6% in year 2000 to 86.7% in 2012 among Thai adults [22]). Since one of the health problems from high sugar consumption is the development of dental caries, it is important to understand from a policy perspective what impact the new SSB tax policy will have on the prevalence of dental caries. Similar studies have been conducted in other countries investigating SSB tax and its impact on dental caries [23–25], while other studies examined SSB tax and its impact on reduced sugar consumption, energy intake and obesity [26–29].

The Thai tiered SSB tax in 2017 adopted a mix tax rate system with both *ad valorem* and specific rate [30]. The *ad valorem* portion of the tax is calculated from the suggested retail price, while the specific tax component depends on the sugar content. The specific tax is generated as such: sugar content higher than 14 g per 100 ml prompts high tax, sugar content of 8–14 g per 100 ml prompts moderate tax, sugar content of 6–8 g per 100 ml prompts low tax and sugar content lower than 6 g per 100 ml is not taxed at all. Upon implementation, this tax rate will increase every 2 years until 2023 [30]. SSB that will be subject to the SSB tax include packaged and ready to drink products such as carbonated soft drinks with added sugar, fruit and vegetable juices, coffee, tea, energy drinks and beverage concentrates for vending machines. With the SSB tax policy, it is expected that sugar consumption among the Thai population will reduce, eventually leading to a reduction in the prevalence of obesity, Type 2 diabetes and tooth decay [25]. However, the SSB tax is not applicable to other non-packaged or non-ready to drink beverages such as herbal drinks, tea and coffee in coffee shops and street vendors. Aside from Thai desserts and snacks, sweet drinks from coffee shops and street vendors are common sources of added sugar consumed by the Thai population.

Oral health issues can be conceptualized as a complex system linked to multiple factors [31, 32], and these factors are diverse, myriad, context dependent and constantly changing. Failing to address that complexity can lead to an inadequate consideration of the dynamic relationships, diverse perspective and invisible boundaries that influence both SSB tax policy and oral health outcomes. Traditional epidemiological and economic approaches may be limited as they may not account for the non-linear and complex relationship among variables [31, 33]. A model-based study in Germany showed that a 20% increase in SSB tax was associated with a reduction in caries, especially among the young and low income population [25]. However, the effect of SSB tax on sugar consumption and dental caries is far from straightforward. Thus, it is uncertain if a tax increase will translate into lower sugar consumption and better oral health outcomes. Understanding the causal mechanisms through which the SSB tax will translate into lower sugar consumption and improved oral health is important in identifying a leverage point for interventions. Dental caries can also be prevented through behavioral change interventions, such as sugar consumption reduction, oral hygiene practices and the utilization of dental health care services. The aim of the study is to explore the expected impact of the new SSB tax policy and other alternative policies on oral health outcomes in Thailand, as measured through the prevalence and distribution of dental caries among the Thai population.

## Methods

The systems science methodology of system dynamics was used [34, 35]. System dynamics modelling is a process of problem identification, causal hypothesis generation, diagramming the proposed causal relationships, translation of qualitative hypothesis into quantitative simulation, reliability testing and policy analysis [34, 35]. Qualitative system dynamics focuses on the use of causal hypothesis generation tools, such as causal loop diagram, to articulate and visualize our understanding of the complex relationships, dynamics, and interconnectedness between interacting variables that are affecting or are affected by the issue of interest. Meanwhile, quantitative system dynamics models consist of interacting sets of differential and algebraic equations developed from the translation of the qualitative causal loop diagram to a quantitative stock and flow model from a broad range of relevant empirical data to capture dynamic interrelationship [34–36]. The systems modelling approach has been used to analyze various oral health problems, including modelling oral healthcare service system in the Netherlands [37], participation in oral health promotion in New York [38] and caries reductions and cost savings from early childhood caries intervention in the United States [39, 40].

In order to gain a better understanding of the SSB tax policy and its likely impact on sugar consumption, an extensive literature review was conducted, leading to the development of guiding questions for an in-depth interview with stakeholders including public health policy makers, consumer foundation representatives, the Thai beverage association and tax excise department officers and health economists. Using insights from the in-depth interviews with stakeholders, a participatory stakeholders’ engagement was organized to build stakeholder consensus, develop a deeper understanding, and map out the causal and dynamic relationships between SSB tax, sugar consumption and oral health outcomes. A detailed description of the in-depth interview with stakeholders and the participatory stakeholder engagements is reported in the reference as cited [41].

Briefly, a key informant interview was conducted in October 2016, and the key informants were identified through a stakeholder analysis. ‘Stakeholders’ herein refer to a group of knowledgeable individuals with complex personal and institutional experiences, beliefs and perceptions that can affect or are affected by the proposed SSB tax policy. A total of 7 informants were interviewed for 30 to 60 min in-person individually, using a semi-structured, open-ended questionnaire. The questionnaire covered a broad list of issues including sugar content in SSB and sugar consumption, general and oral health outcomes as a consequence of sugar consumption, concerns regarding the proposed SSB tax, and expected barriers and consequences. The interview was audio-recorded and transcribed. The purpose of the key informant interviews were to gain a deeper understanding of the perspectives of the multi-sector stakeholders on the proposed SSB tax, and its plausible consequences on sugar consumption and oral health outcomes. Following the key informant interviews, a participatory stakeholder engagement via Group Model Building (GMB) was conducted in February 2017. Details of the GMB process can be found in the references [32]. GMB refers to a system dynamics model building process, in which stakeholders are deeply and actively involved in the process of qualitative model construction through the exchange, assimilation, and integration of mental models into holistic system description [42]. Two GMB sessions lasting 3 h each were conducted with 10 stakeholders, including those involved in the key informant interviews. The outcome from the stakeholder engagement was a qualitative system dynamics model that describes the complex dynamic interrelationships of SSB tax policy, sugar consumption and dental caries.

A simulation model was developed following the stakeholders’ engagement to simulate the bahaviour over time of key outcome variables, and presented to the stakeholders to verify the model structure and assumptions underlying postulated causal relationships. Both causal loop diagram and stock and flow diagram were developed using Vensim DSS version 6.4 (Ventana Inc). After verification, the model was parameterized using a series of empirical data. When data was not available, estimates from experts were used.

### Simulation model structure

The dental caries simulation model projects the prevalence and distribution of dental caries severity among the Thai population 15 years and older. The simulation model consists of three interconnected sub-models: caries prevalence sub-model (SM1), dental service utilization sub-model (SM2), and oral health behavior sub-model (SM3) (Appendix A).

### Caries prevalence sub-model (SM1)

**Fig. 1:**
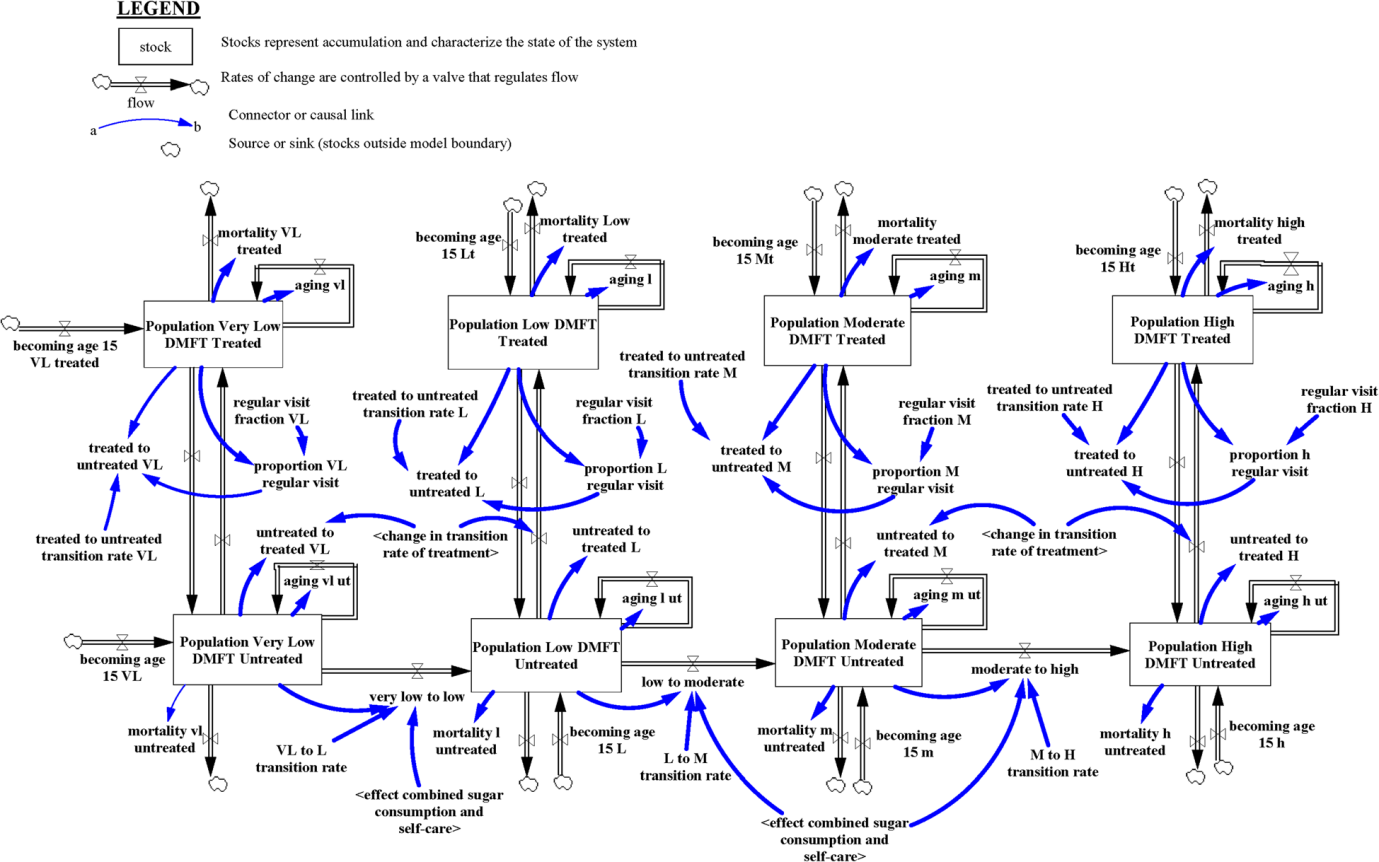
Caries prevalence sub-model

The caries prevalence sub-model (SM1)—see Fig. 1—projects the oral health status (OHS), measured herein by Decayed-Missing-Filled Teeth (DMFT) (Appendix B), among the Thai population 15 years and older. OHS was divided into four categories (see Table 1)—i.e. Very Low (VL) DMFT, Low (L) DMFT, Moderate (M) DMFT, and High (H) DMFT—based on WHO classification [43]. The WHO criteria of level of dental caries experience disaggregates the DMFT classification into two different age cohorts: children 12 years and adults of 35 to 44 years of age. For the purpose of this research, we used the DMFT classification of children 12 years of age for the Thai population 15–34 years of age; while the adult classification of 35 to 44 years of age was used for the Thai population 35 years and older. In addition, each OHS was further divided into two groups: treated and untreated dental caries. The treated population are individuals needing no normative treatment, or dental prosthesis, while the untreated population are individuals with dental treatment needs.

**Table 1:** WHO severity criteria for level of dental caries experience in permanent dentition [43]

Oral health status (Dental caries experience)	Children 12 years DMFT (Teeth/person)	Adult 35–44 years DMFT (Teeth/person)
Very low (VL)	< 1.2	< 5.0
Low (L)	1.2–2.6	5.0–8.9
Moderate (M)	2.7–4.4	9.0–13.9
High (H)	> 4.5	> 13.9

SM1 assumes progressive and unidirectional transitions across OHS. Transition rates across OHS groups were estimated using calibration, which is the process of adjusting model parameters to obtain an outcome comparable to available data (distribution of oral health outcomes). The transitions within each OHS is bidirectional, meaning an individual can move from treated to untreated and vice versa. The conceptualization of SM1 began with the instantiation of the simulation model, and the Thai population 15 years and older was distributed into the four OHS. The population in each OHS increases as (a) the population becoming 15 years old flows into that OHS and the new entrants (15 years old) are distributed across the four OHS (i.e. Very Low (VL) DMF, Low (L) DMFT, Moderate (M) DMFT, and High (H) DMFT); (b) when individuals in one OHS transition to another. Similarly, the numbers of population in each OHS decreases (a) via deaths and (b) when individuals in one OHS transition from that OHS to another OHS. SM1 accounts for aging within each health status to ensure that at the end of every year, the surviving population in each OHS transitions to the appropriate age cohort, since age is an important factor for death. Transitions from untreated to treated status are influenced by the change in uptake rate of treatment, which is derived from dental service utilization sub-model (SM2). Transitions from treated to untreated status are influenced by the regular dental visits fraction, which was calculated from the numbers of population who visited the dentists in the past year prior to the national oral health survey. Data from the Thai national oral health survey in 2000–2001 (S1), 2006–2007 (S2) and 2012 (S3) was used as the main data source.

### Dental service utilization sub-model (SM2)

**Fig. 2:**
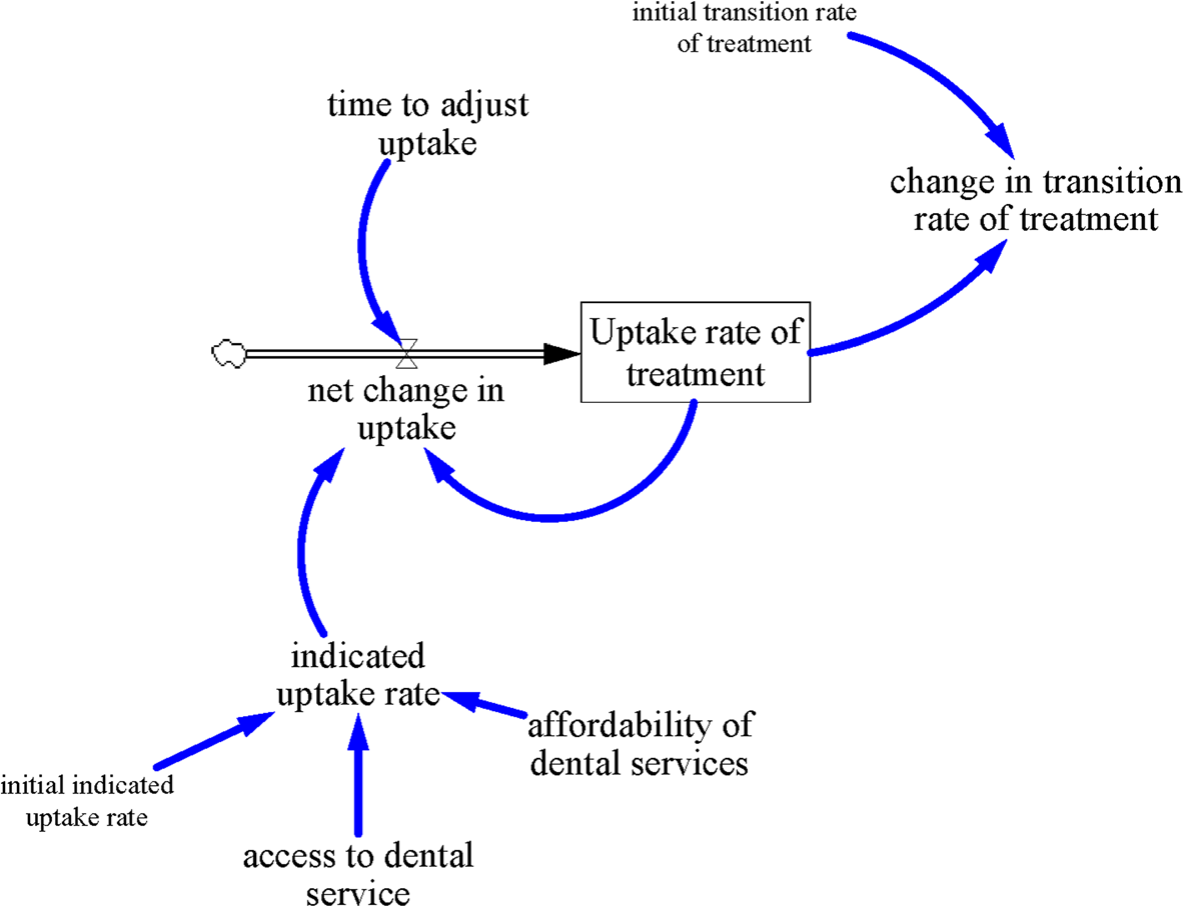
Dental service utilization sub-model

The dental service utilization sub-model (SM2) (Fig. 2) simulates the dynamics of dental services use. Dental services refer to care services for the purpose of the maintenance of healthy teeth, and includes examination and diagnosis of dental problems, restorative dentistry, periodontics, extraction of teeth under local anesthesia and curettage of infected socket, and preventive dentistry and oral health education. Here, the proportion of people using dental services (uptake rate of treatment) is affected by the number of new people using the services (herein referred to as net change in uptake rate). Net change in uptake rate (referring to the difference between the number of new people seeking dental treatment minus the number of people leaving dental treatment) is herein determined by the most current data on uptake rate (herein referred to as indicated uptake rate of dental services), last measured uptake rate of dental services (herein referred to as uptake rate of dental services), and time to adjust uptake rate of dental services. Indicated uptake rate of dental services is determined by access to dental services and affordability of dental care. Access to dental services focuses on availability of dental services, while affordability of dental services focuses on the ability of the population to pay for dental care services. The ratio of population per dental personnel, including dentists and dental nurses, was used as a proxy for access to dental services. It was assumed that increased access to dental care would increase the treatment uptake rate. Furthermore, affordability of dental health services is assumed to vary across socio-economic group. Low-income individuals are assumed to struggle with out-of-pocket costs of dental services, whereas high-income individuals are assumed to have no such problem. Accessibility and affordability of dental services are represented in the model as policy variables that could be further explored due to its potential impact on uptake rate of dental services.

### Oral health behavior sub-model (SM3)

**Fig. 3:**
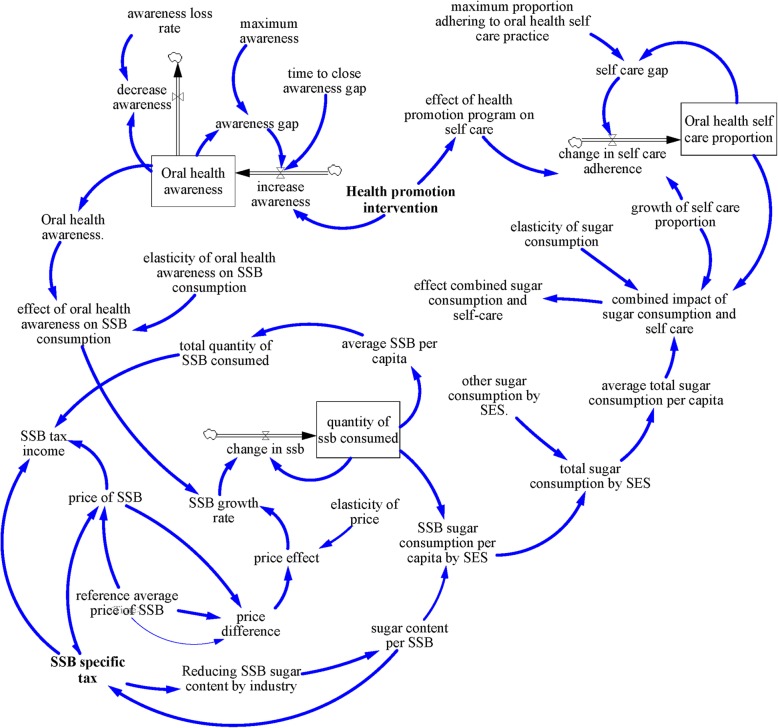
Oral health behavior sub-model

The oral health behavior sub-model (SM3) (see Fig. 3) models the dynamic relationship between oral health awareness, sugar consumption, and oral health self-care practice. Oral health awareness is the prevalence of oral health knowledge (i.e. awareness of oral diseases or risk factors or preventive measures) among the population. For the purpose of this research, the proportion of the Thai population who receive oral health knowledge from television, social media, family and friends were used as a proxy for oral health awareness. In order to simplify the model structure for oral health awareness, we assumed a maximum level of oral health awareness achievable. Hence a maximum oral health awareness rate is compared with current oral health awareness rate to determine the oral health awareness gap. Any gap in oral health awareness is assumed to be closed by health promotion campaigns over time. Furthermore, loss in oral health awareness over time was represented, as determined by the loss rate of awareness.

Sugar consumption, which measures the average added sugar consumption, was divided into sugar consumed from sugar sweetened beverages (SSB) and other sources of sugar, including that consumed from coffee shops, street beverages, desserts and other food, and home sugar consumption. The SM3 model assumes that SSB consumption is affected by income; higher income individuals are assumed to consume more SSB relative to the lower-income individuals. Based on evidence from other studies [29], we postulated that SSB tax is likely to have less impact on the high-income group compared to the low-income group. In order to estimate the quantity of sugar intake from SSB, the quantity of SSB consumed was multiplied by the average sugar content per SSB. Lastly, current sugar consumption was compared to initial sugar consumption to derive relative sugar consumption to estimate the change in sugar consumption.

Self-care practice referred to the proportion of the population who brush their teeth at least twice a day with fluoride toothpaste. It was modeled as a stock which changes over time. Net change in self-care practice is determined through the gap between current self-care practice, maximum self-care practice and health promotion campaigns. As health promotion increases, self-care is assumed to increase simultaneously (Table 2).

**Table 2:** Model parameters

Parameters	Values		Unit	Source
**Dental caries sub-model (SM1)**	Age 15–34	Age 35+		
Regular visit fraction			Dimensionless/ year	[44]
very low DMFT [female]	0.229	0.148		
very low DMFT [male]	0.191	0.170		
low DMFT [female]	0.306	0.357		
low DMFT [male]	0.308	0.385		
moderate DMFT [female]	0.521	0.643		
moderate DMFT [male]	0.462	0.333		
high DMFT [female]	0.571	0.332		
high DMFT [male]	0.583	0.215		
Treated to untreated transition			Dimensionless/ year	Expert estimation
very low DMFT	0.4	0.7		
low DMFT	0.78	0.58		
moderate DMFT	0.78	0.34		
high DMFT	0.7	0.75		
^a^Very Low To Low transition rate	0.063 (0.0504–0.0756)		Dimensionless/year	Model calibration
^a^Low To Moderate transition rate	0.066 (0.0528–0.0792)			
^a^Moderate To High transition rate	0.063 (0.0504–0.0756)			
**Dental health utilization sub-model (SM2)**				
Time to adjust uptake	1		Year	Expert estimation
Initial uptake rate	VL 0.384, L 0.066, M 0.041, H 0.075		Dimensionless	[44]
**Oral health behaviour sub-model (SM3)**				
Reference average price of SSB	Report on product price, 2000–2018		Thai Baht	[45]
^a^Demand price elasticity	Low income −1.46 (− 1.168-1.752) High income − 0.39 (− 0.312–0.468)		Dimensionless	[46]
Average sugar content per SSB	0.15 (0.5–0.9)		Kg/litre	[47]
Other sugar consumption by SES [low income, high income]	Report from 2000 to 2015 and extrapolation after 2015 from 4y % change moving average			[7]
Initial self-care adherence	0.529		Dimensionless	[44]
^a^Elasticity of sugar consumption	0.6 (0.4–0.72)		Dimensionless	Expert estimation
^a^Percent reduce SSB sugar	0.8 (0.5–0.9)		Dimensionless	Expert estimation

^a^Parameters used for sensitivity analysis

### Policy simulation

#### Base-case

This scenario represents the situation before the SSB tax was implemented. All parameters and key variables remain unchanged over the simulation run. This serves as a reference point for comparing four other scenarios.

#### SSB tax

This scenario implements the proposed SSB tax policy from 2018 to 2040. Table 3 shows the SSB tax in Thai Baht (Thailand currency) by SSB sugar content and the year of implementation. As indicated in Table 3, SSB with sugar content between 0 and 5.99 g/100 ml attracts no tax. However, SSB with sugar content between 6.0–8.0 g/100 ml attracts SSB tax that increases from 0.1 Thai Baht to 1 Thai Baht by 2040. Likewise, SSB with sugar content between 8.01–10.0 g/100 ml will attract SSB tax that rises from 0.3 Thai Baht to 3 Thai Baht by 2040. SSB with sugar content of 10.01–14.0 g/100 ml and 14.01–18.0 g/100 ml will attract SSB tax that increases from 0.5 Thai Baht and 1 Thai Baht, respectively to 5 Thai Baht by 2040. Lastly, SSB with sugar content above 18 g/100 ml will attract SSB tax that increases from 1 Thai Baht to 5 Thai Baht by 2040.

**Table 3:** SSB tax policy

	Specific tax			
Sugar Content (g/100 ml)	2018–2019 (Thai Baht)	2021–2022 (Thai Baht)	2023–2024 (Thai Baht)	2025–2040 (Thai Baht)
0–5.99	0	0	0	0
6.0–8.0	0.1	0.1	0.3	1
8.01–10.00	0.3	0.3	1	3
10.01–14.00	0.5	1	3	5
14.01–18.00	1	3	5	5
more than 18.00	1	5	5	5

#### Aggressive policy scenario

This scenario consists of: (a) SSB tax policy scenario, (b) non-SSB sugar consumption scenario, and (c) dental care services use scenario. The SSB tax policy scenario is exactly the same as the SSB tax policy with different SSB tax rates for different SSB sugar content in years 2018 to 2020, 2021 to 2022, 2023 to 2024, and 2025 to 2040. However, the non-SSB sugar consumption scenario assumes that non-SSB sugar consumption will decrease 80% in comparison with the base-case sugar consumption from 2018 to 2040. Thus, non-SSB sugar consumption is assumed to decrease from 22.35 kg/person/year to 4.47 kg/person/year for low income individuals and 33.01 kg/person/year to 6.6 kg/person/year for high income individuals in 2018 (the base year for the policy implementation). Meanwhile, that for 2040 is assumed to decrease from 28.04 kg/person/year to 5.6 kg/person/year for low income individuals, and 43.15 kg/person/year to 8.63 kg/person/year for high income individuals. Likewise, the dental care services use scenario assumes that uptake rate of dental care services will increase 50% from 39 to 50.85% in 2018 (the base year for the policy implementation) and remain unchanged to 2040.

### Sensitivity analysis

Sensitivity analysis performed on all the scenarios proposed in the study aimed to observe the effect of parameter changes on the main outcomes of interest (population in each DMFT group and sugar consumption). Using multivariate sensitivity analysis, the parameters that were deemed to be sensitive to the outcomes of interest were varied by ±20% and impact on the outcomes were measured at 95% confidence level. Table 2 indicates the list of parameters included in the sensitivity analysis.

## Results

Under the base-case scenario, the prevalence of dental caries, among the Thai population 15 years and older is projected to increase from 61.3% in 2010 to 74.9% by 2040. Implementation of SSB tax policy is expected to decrease the prevalence of dental caries by only 1% by 2040, whereas the aggressive policy is projected to decrease prevalence of dental caries by 21% by 2040. The mean DMFT values for individuals 15–34 years, under the base-case is projected to increase from 2.42 in 2010 to 2.60 by 2040. Similarly, the mean DMFT value under the SSB tax policy, by 2040 is 2.58 (representing 0.76% relative to the base-case), while that for the aggressive policy is 2.22, (representing 14.6% reduction relative to the base-case). For those 35 years and older, the mean DMFT value is estimated to increase from 2.22 in 2010 to 2.92 by 2040. By 2040, the mean DMFT for the SSB policy is projected to be 2.89 (which is 1.03% reduction), whereas that for the aggressive policy is 2.15, representing 26.3% reduction.

Table 4 shows the projected distribution of the population by DMFT severity. For the base-case scenario, the population with low DMFT is projected to decrease from 9.95 million in 2010 to 9.76 million (9.63 million-9.89 million) by 2040, representing a reduction of 2%. However, the population with moderate DMFT is projected to increase 18% from 8.37 million in 2010 to 9.87 million (9.73 million-9.96 million) by 2040; whereas those with high DMFT is also projected to increase 58% from 12.56 million in 2010 to 19.79 million (19.58 million-20 million) by 2040. In 2040, the elderly population (65 years and older) is projected to constitute 21, 27 and 39% of the individuals with low, moderate and high DMFT, respectively. In addition, the proportion of untreated dental caries is projected to remain high—around 91%—among those with low, moderate and high DMFT.

**Table 4:** Projected population by dental caries status (millions)

Outcomes		2010	2020	2030	2040	% change 2010–2040	% change to base-case
Base-case	Very low DMFT	19.52 (19.43–19.61)	17.70 (17.56–17.85)	15.52 (15.35–15.70)	13.20 (13.02–13.38)	−31%	–
	Low DMFT	9.95 (9.86–10.05)	10.94 (10.82–11.06)	10.73 (10.60–10.86)	9.76 (9.63–9.89)	− 2%	–
	Moderate DMFT	8.37 (8.30–8.43)	9.52 (9.42–9.61)	10.09 (9.98–10.20)	9.87 (9.73–9.96)	18%	–
	High DMFT	12.56 (12.51–12.61)	15.21 (15.10–15.31)	17.76 (17.60–17.92)	19.79 (19.58–20.00)	58%	–
SSB Tax Policy	Very low DMFT	19.52 (19.43–19.61)	17.70 (17.56–17.85)	15.73 (15.56–15.91)	13.65 (13.47–13.83)	−30%	3%
	Low DMFT	9.95 (9.86–10.05)	10.94 (10.82–11.06)	10.76 (10.63–10.88)	9.88 (9.75–10.01)	−1%	1%
	Moderate DMFT	8.37 (8.30–8.43)	9.52 (9.42–9.61)	10.06 (9.96–10.17)	9.83 (9.72–9.94)	18%	−0.1%
	High DMFT	12.56 (12.51–12.61)	15.20 (15.10–15.31)	17.54 (17.38–17.70)	19.24 (19.03–19.44)	53%	−3%
Aggressive Policy	Very low DMFT	19.52 (19.43–19.61)	18.86 (18.73–18.99)	20.91 (20.79–2104)	21.49 (21.37–21.62)	10%	63%
	Low DMFT	9.95 (9.86–10.05)	10.91 (10.80–11,03)	11.46 (11.35–11.56)	11.48 (11.38–11.58)	15%	18%
	Moderate DMFT	8.37 (8.30–8.43)	9.34 (9.25–9.43)	9.37 (9.30–9.44)	8.92 (8.86–8.98)	7%	−9.4%
	High DMFT	12.56 (12.51–12.61)	14.26 (14.17–14.35)	12.35 (12.27–12.43)	10.70 (10.63–10.77)	−15%	−46%

By 2040, the implementation of the SSB tax policy is projected to nominally increase the individuals with low DMFT by 1% compared to the base-case scenario; individuals with moderate and high DMFT are projected to decrease by 0.1 and 3%, respectively, compared to the base-case scenario. Likewise, the proportion of untreated dental caries is projected to remain at 91% for all DMFT groups. Nonetheless, by 2040, the implementation of the aggressive policy is projected to increase the number of individuals with low DMFT by 18% compared to the base-case scenario, while that for moderate and high DMFT are projected to decrease significantly by 9.4 and 46% respectively compared to the base-case scenario. Furthermore, the proportion of untreated dental caries is projected to decrease significantly to 58% for low DMFT, 49% for moderate DMFT, and 62% for high DMFT.

## Discussion

Based on the simulation analysis, the implementation of the proposed tiered SSB tax in Thailand is expected to reduce SSB consumption among both the low- and high- income groups, with the highest impact observed in the longer term due to higher SSB tax rates in later years. This finding is consistent with other studies [48–50]. The main insight from this research suggests that SSB tax policy alone is unlikely to have any meaningful impact on oral health outcomes, as defined by dental caries, unless it is accompanied by a comprehensive policy that aims to reduce total sugar intake from non-tax sugary food sources. Countries where a majority of the sugar consumed are from non-tax sugary food and beverages, especially Asian countries where street food culture is ubiquitous and contributes disproportionately to sugar intake, may find this insight especially relevant. The policy implications of this insights are: (a) countries with a significant proportion of sugar intake from non-tax sources, such as coffee shops, and high sugar content desserts and food from street shops, should design innovative policies to reduce sugar consumption; (b) although over-reliance on SSB tax will increase resources for other public health interventions, it is unlikely to produce the expected health outcomes in dental caries; (c) policies designed to reduce sugar intake from non-SSB sources will require significant stakeholder engagement to increase the likely buy-in from stakeholders.

Although the simulation model presented has substantial implications for policy, it should be interpreted with caution. The main strength of this study is the active engagement of stakeholders in the development and validation of the simulation model, as well as the synthesis of data from various sources to populate the simulation model in order to evaluate the likely impact of the SSB policy and alternative policies on dental caries. Additionally, the simulation model allows policymakers to observe the likely impact of their policies *in-silico* before they are implemented. This research is an exercise to inform policymakers in Thailand of the likely impact of the proposed SSB tax policy on the prevalence of dental caries. This is important because the proposed SSB tax and its impact on sugar consumption and health outcomes (such as dental caries) is a systemic-pluralist problem. It is systemic because the problem lies in the complexity of the system which is often partially observable, probabilistic in nature, evolving over time, and subject to behavioral influences [51]; it is pluralistic because possible solutions to the problem are diverse and might be acceptable by some stakeholders but not to others [51]. Hence, this research exercise could be used by policymakers to engage other stakeholders to discuss policy options, modify model assumptions and structure and consider holistically, the impact of the proposed SSB tax on other health outcomes.

However, one limitation of the study is that a change in the projected population will affect the number of people by DMFT groups. Furthermore, due to lack of longitudinal data, we were unable to estimate and use evidence-based transition rates across different DMFT groups by age, gender and socio-economic groups, leading to a likely under or overestimation of the distribution of the population by DMFT groups.

## Conclusion

In this study, a simulation model was used to evaluate the impact of a proposed tiered SSB tax in Thailand on dental caries among the Thai population. The results suggest that without combining SSB tax with a comprehensive policy that aims to reduce total sugar intake from non-SSB sources, implementing SSB tax alone will have minimal impact on dental caries. The simulation model presented can not only provide policy makers with additional insight to support future oral health policy planning efforts, but also guide the general population towards making healthier choices.

## Data Availability

The datasets used and/or analyzed during the current study are available from the corresponding author on reasonable request.

## Appendix A

### Caries Prevalence sub-Model (SM1)

This sub-model disaggregates the population of Thailand 15 years and older into four oral health status (i.e. population Very Low DMFT, population low DMFT, population moderate DMFT, and population high DMFT). To differentiate between individual treated and untreated, each oral health status was divided into two treatment status (i.e. untreated and treated); as a result, eight oral health status were obtained (population Very Low DMFT untreated, population Very Low DMFT treated, population low DMFT untreated, population low DMFT treated, population moderate DMFT untreated, population moderate DMFT treated, population high DMFT untreated, and population high DMFT treated). The caries prevalence sub-model operates as follows: at the beginning of each year, the population becoming 15 years (which is derived from the variable “population”) which comes from a validated population model; is divided into the eight oral health status. The assumption here is that individuals becoming 15 years could be in any one of the eight oral health status, thus individuals becoming 15 years is divided into eight groups (i.e. becoming age 15 very low untreated, becoming age 15 very low treated, becoming age 15 low untreated, becoming age 15 low treated, becoming age 15 moderate untreated, becoming age 15 moderated treated, becoming age 15 high untreated, and becoming age 15 high treated). The caries prevalence model assumes that individuals with untreated dental caries can progress from one oral health status to the other, while individual treated individual are assumed remain in the same oral health status; and that transition is unidirectional (people can only progress to the worse oral health status). Thus, population very low DFMT untreated is assumed to progress to population low DMFT untreated over time; and population low DMFT untreated is assumed to progress to population moderate DMFT untreated. Lastly, population moderate DMFT untreated is assumed to progress to population high DMFT untreated over time. The progression from one oral health status to the other is determined by transition rate and the effect of combined sugar consumption and self-care. The population in each oral health status is further disaggregated by single age cohort (age 15 to age 100 and over). The aging process is represented in the model by the variables (aging vk ut, aging vl, aging l ut, aging l, aging m ut, aging m, aging h ut, and aging h). the aging process works as follows, ag the end of each year, the population in each cohort is shifted to the next age cohort to account for aging (thus individuals in cohort age 15 will shift to age 16, while those in age 16 will shift to age 17 until age 100 which is the last age cohort). The movement of individuals from untreated to treated is determined by regular visit fraction (which represent the proportion of population untreated who start receiving regular dental care services). Likewise, individuals move from treated to untreated oral health status when they stop receiving dental care services.

### Dental service utilization sub-model (SM2)

The dental service utilization sub-model simulates the uptake rate of dental treatment. Uptake rate dental treatment increase by net change in uptake rate—which is the difference between individual who are taking up new dental treatment and those leaving dental treatment (attrition). The net change in uptake of treatment is affected by indicated uptake rate—which is determined by affordability of dental services, access to dental services and initial indicated uptake rate. As uptake rate of treatment increases, the transition rate from untreated to treated is assumed to change over time.

### Oral health behavior sub-model (SM3)

The oral health behavior sub-model simulates the impact of health promotion intervention on oral health self-care and oral health awareness, and its subsequent impact on SSB consumption. In the model, health promotion intervention is assumed increase oral health awareness, while oral health awareness decreases due to awareness loss rate. As oral health awareness, and price of SSB increase, it is assumed to affect the SSB growth rate—the increase in oral health awareness and SSB price will decrease the increase in SSB growth rate. As SSB growth rate decreases, quantity of SSB consumed is assumed to decrease, leading to lower average SSB per capita and total quantity of SSB consumed. The price of SSB is affect by reference average price of SSB and SSB specific tax. The price difference is the difference between reference average price before the SSB tax and price of SSB after SSB tax. As price difference increase, the effect of price on SSB consumption is assumed to increase, thus decreasing the amount of SSB consumed. In addition, the introduction of SSB specific tax is assumed to decrease the sugar content in SSB, thus eventually decreasing the total sugar consumption. As SSB sugar consumption per capita by SES (socio-economic status) decreases, total sugar consumption by SES is assumed to decrease, which together with change in oral health self-care is assumed to impact of transition to worse oral health status.

## Appendix B

### Calculation of DMFT [43]

**DMFT and DMFS** measures dental caries severity or intensity in an individual. DMFT and DMFS are means to numerically express the caries severity and are obtained by calculating the number of (a) Decayed (D), (b) Missing (M), (c) Filled (F) teeth (T) or surfaces (S). It is thus used to get an estimation illustrating how much the dentition until the day of examination has become affected by dental caries. It is either calculated for 28 (permanent) teeth, excluding 18, 28, 38 and 48 (the “wisdom” teeth) or for 32 teeth (The Third edition of “Oral Health Surveys - Basic methods”, Geneva 1987, recommends 32 teeth). Thus: (a) how many teeth have caries lesions (incipient caries not included)? (b) how many teeth have been extracted? (c) how many teeth have fillings or crowns? The sum of the three figures forms the DMFT-value. For example: DMFT of 4–3-9 = 16 means that 4 teeth are decayed, 3 teeth are missing and 9 teeth have fillings. It also means that 12 teeth are intact. Note: If a tooth has both a caries lesion and a filling it is calculated as D only. A DMFT of 28 (or 32, if “wisdom” teeth included) is maximum, meaning that all teeth are affected. A more detailed index is DMF calculated per tooth surface, DMFS. Molars and premolars are considered having 5 surfaces, front teeth 4 surfaces. Again, a surface with both caries and filling is scored as *D. maximum* value for DMFS comes to 128 for 28 teeth. For the primary dention, consisting of maximum 20 teeth, the corresponding designations are “deft” or “defs”, where “e” indicates “extracted tooth”.

## Abbreviations

DMFT: Decayed-Missing-Filled Teeth
H: Hight DMFT
L: Low DMFT
M: Moderate DMFT
OHS: Oral health status
S1: Year 2000–2001
S2: Year 2006–2007
S3: Year 2012
SM1: Caries prevalence sub-model
SM2: Dental service utilization sub-model
SM3: Oral health behavior sub-model
SSB: Sugar-sweetened beverage
VL: Very Low DMFT
